# Effects of a Preventive Mental Health Curriculum Embedded Into a Scholarly Gaming Course on Adolescent Self-Esteem: Prospective Matched Pairs Experiment

**DOI:** 10.2196/48401

**Published:** 2023-12-06

**Authors:** Christopher Jenson, Sharon Fitzgerald Wolff, Libby Matile Milkovich

**Affiliations:** 1 Diagnosing Education, LLC, Overland Park, KS, United States; 2 University of Kansas Medical Center, Kansas City, KS, United States; 3 Children's Mercy Hospital and Clinics, Kansas City, MO, United States; 4 School of Medicine, University of Missouri, Kansas City, MO, United States

**Keywords:** mental health, adolescent, scholastic gaming, self-esteem, school mental health, school, youth, student, students, adolescents, gaming, serious game, serious games

## Abstract

**Background:**

Positive self-esteem predicts happiness and well-being and serves as a protective factor for favorable mental health. Scholarly gaming within the school setting may serve as a channel to deliver a mental health curriculum designed to improve self-esteem.

**Objective:**

This study aims to evaluate the impact of a scholarly gaming curriculum with and without an embedded preventive mental health curriculum, Mental Health Moments (MHM), on adolescents’ self-esteem.

**Methods:**

The scholarly gaming curriculum and MHM were developed by 3 educators and a school-based health intervention expert. The scholarly gaming curriculum aligned with academic guidelines from the International Society for Technology Education, teaching technology-based career skills and video game business development. The curriculum consisted of 40 lessons, delivered over 14 weeks for a minimum of 120 minutes per week. A total of 83 schools with previous gaming engagement were invited to participate and 34 agreed. Schools were allocated to +MHM or –MHM arms through a matched pairs experimental design. The –MHM group received the scholarly gaming curriculum alone, whereas the +MHM group received the scholarly gaming curriculum plus MHM embedded into 27 lessons. MHM integrated concepts from the PERMA framework in positive psychology as well as the Collaborative for Academic, Social, and Emotional Learning (CASEL) standards in education, which emphasize self-awareness, self-management, social awareness, relationship skills, and responsible decision-making. Participants in the study were students at schools offering scholarly gaming curricula and were enrolled at recruitment sites. Participants completed a baseline and postintervention survey quantifying self-esteem with the Rosenberg Self-Esteem Scale (score range 0-30). A score <15 characterizes low self-esteem. Participants who completed both baseline and postintervention surveys were included in the analysis.

**Results:**

Of the 471 participants included in the analysis, 235 received the –MHM intervention, and 236 received the +MHM intervention. Around 74.9% (n=353) of participants were in high school, and most (n=429, 91.1%) reported this was their first year participating in scholarly gaming. Most participants were male (n=387, 82.2%). Only 58% (n=273) reported their race as White. The average self-esteem score at baseline was 17.9 (SD 5.1). Low self-esteem was reported in 22.1% (n=104) of participants. About 57.7% (n=60) of participants with low self-esteem at baseline rated themselves within the average level of self-esteem post intervention. When looking at the two groups, self-esteem scores improved by 8.3% among the +MHM group compared to no change among the –MHM group (*P*=.002). Subgroup analyses revealed that improvements in self-esteem attributed to the +MHM intervention differed by race, gender, and sexual orientation.

**Conclusions:**

Adolescents enrolled in a scholarly gaming curriculum with +MHM had improved self-esteem, shifting some participants from abnormally low self-esteem scores into normal ranges. Adolescent advocates, including health care providers, need to be aware of nontraditional educational instruction to improve students’ well-being.

## Introduction

Self-esteem is an important marker of overall adolescent well-being. Positive self-esteem predicts happiness, academic performance, and resiliency among other favorable outcomes, whereas low self-esteem contributes to anxiety and depression [1,2]. Because self-esteem can be either a risk factor or a protective factor for mental health challenges, it is critically important to target with supportive initiatives.

Adolescence is a pivotal time to develop positive self-esteem. A strong sense of self-worth propels adolescents to explore new opportunities and may help navigate feelings of inadequacy when experiencing common challenges at this age, such as friend group transitions, feelings of awkwardness, and unexpected emotional lability [3]. The benefits of self-esteem have been especially relevant since 2000 when the prevalence of anxiety, depression, and suicidal ideations began to increase in adolescents, worsening during the COVID-19 pandemic, and remaining slow to improve despite the end of COVID-19 restrictions [4-7].

One cohort of adolescents, high-volume technology users, are at increased risk for low self-esteem [8-10]. Multiple hypotheses exist for this relationship, including comparing self to social media standards and the social isolation that can occur with excessive video gaming [11,12]. In contrast, several research inquiries suggest that interactive technology use can be helpful [13,14]. One primary reason is that technology can serve as a motivator for behavioral intervention and contribute to the promotion or reduction of certain behaviors [15]. For example, in the educational space, interactive websites and captivating software apps can ignite student engagement, improve academic performance, and serve as a reward for completed learning objectives [16].

A more extensive example of using technology to positively shape behavior is scholarly gaming. Scholarly gaming is an academic course in secondary schools that leverages student excitement around video gaming to teach technology-based career skills, project-driven teamwork, and accountability for adolescents. However, there appears to be a gap that scholarly gaming has yet to fill—supporting the self-esteem of student participants. Many of the students who enroll in scholarly gaming classes may not be well connected to school life outside of their interest in technology and gaming, putting them at risk for feelings of isolation [17].

Our intervention offered a scholarly gaming curriculum that embedded opportunities to develop positive self-esteem within the academic instruction, serving as a vessel to deliver a mental health curriculum; offered schools an opportunity to improve the emotional well-being of adolescents at baseline; and promoted an inclusive extracurricular activity for interested students, boosting feelings of self-worth and confidence [18]. With these design elements, scholarly gaming could offer a practical and affordable approach to student mental health support, which many experts in education desire [19,20].

The purpose of this study is to evaluate the impact of scholarly gaming with and without an embedded mental health curriculum, known as Mental Health Moments (MHM), on the self-esteem of adolescents. We hypothesized that students participating in a scholarly gaming curriculum with embedded MHM (+MHM) would show greater improvement in self-esteem compared to the same scholarly gaming curriculum without the MHM (−MHM). To evaluate the hypothesis, we quantitatively assessed the self-esteem of adolescents at baseline and post intervention (+MHM or –MHM).

## Methods

This prospective study compared baseline and postintervention self-reported self-esteem scores from adolescents participating in +MHM and –MHM. This study occurred from December 2021 through May 2022.

### Setting and Participants

Recruitment for this study occurred at secondary schools in Canada and the United States. Eligible schools were identified by reviewing prior enrollment in school-sponsored gaming events, demonstrating that the school had the tech resources to support a scholarly gaming course. All schools that had participated in school-sponsored gaming events (N=83 middle and high schools in North America) were invited via email to participate in the study. A total of 34 schools agreed to participate, representing more than 30 independent districts across the United States (11 states) and Canada (1 province). There was no statistical difference between the schools that enrolled versus those that declined when comparing student body size, public versus private, Title 1 status, and poverty rates (Multimedia Appendix 1). There was a slight geographic difference between schools who agreed and declined to participate, with schools in the Midwest (55%) and West (55%) being more likely to enroll than schools in the Southwest (33%), Northeast (20%), or Southeast (7%; *P*<.001; Table S1 in Multimedia Appendix 1).

The most common reason for declining enrollment in the study was faculty preference, as some teachers did not want to deviate from pre-existing lesson plans. A total of 17 schools each were allocated to the intervention (+MHM) and control (–MHM) arms.

Participant eligibility included enrollment in a course that provided the –MHM or +MHM scholarly gaming curriculum at a participating school, aged 12-18 years, and a class schedule that allowed engagement with the study-approved curriculum for 120 minutes each week. No student was required to participate, and students could withdraw at any time from the survey process. There was no compensation or benefits in kind for participation.

### Ethical Considerations

Participating schools considered their local legislation and institutional requirements before joining the study. Additionally, scholarly gaming teachers provided all eligible participants with a written informed assent/consent form to share with their guardians. The institutional review board at Children’s Mercy Hospital and Clinics (Kansas City, MO) approved the secondary analysis of data (STUDY00002344) collected by the study investigators who designed the +MHM and −MHM programs.

### Study Design and Allocation

In this prospective study, participating schools were allocated to +MHM and –MHM arms through a matched pairs experimental design, balancing the number of students enrolled in the school, Title 1 status, public versus private, and geography.

### Interventions

#### Teacher Training

All teachers received the following resources at no cost: access to either the +MHM or −MHM curriculum guides on the web, depending on their study allocation; paper versions of the curriculum guides as a backup; student handouts for applicable lessons; and three video tutorials. The curriculum guides included scripted lessons, along with standardized presentation materials and supplements for each lesson. The video tutorials explained how to use the scholarly gaming curriculum consistently and follow the data collection process, and emphasized the need for a standardized experience throughout the study. Finally, teachers were informed that the study sought to measure the impact of a new curriculum on self-esteem over time but were blinded as to whether they were providing +MHM or –MHM.

#### Standard Curriculum (–MHM)

The scholastic gaming curriculum included 40 lessons over 14 weeks for a minimum of 120 minutes per week. The goal of the curriculum was to teach students meaningful skills and knowledge needed to work in the digital gaming industry. Examples include project management, graphic design, basic coding, marketing, maximizing team performance, and career opportunities linked to gaming. The curriculum, designed by multiple educators and a school-based health intervention expert, was organized into units, daily lessons, and timed projects. The –MHM curriculum content aligned with the International Society for Technology in Education (ISTE) benchmarks. Lessons were led by the teacher and provided a standardized experience through prewritten lesson plans (within the curriculum) for teachers to follow. Each lesson plan engaged study participants with prescriptive teacher dialogue and premade resources (eg, worksheet or infographic) created by the curriculum writers. The research team verified with the participating schools that the scholastic gaming teacher held the appropriate professional licensure and certification to teach secondary school in that state/province.

#### Standard Curriculum With Embedded Mental Health Moments (+MHM)

Participants allocated to the +MHM group received the same scholastic gaming curriculum and teacher training as those in the –MHM group but with the addition of +MHM embedded into 27 of the 40 scholarly gaming curriculum lessons. To make room for the embedded +MHM, 10 minutes of student work time was removed from the lesson plan. There were no other changes made. +MHM was designed by the same team of professionals as –MHM and aligned with the previously mentioned ISTE standards and validated frameworks in education and psychology that support favorable mental health [21,22]. Additionally, all scholarly gaming teachers who provided +MHM met the same credentialing standards as the –MHM faculty.

The primary goal of +MHM was to promote positive self-esteem in participants, growing confidence in their worth and abilities. +MHM accomplished this goal by linking elements of the tech-based academic lesson to one of three premade student experiences (discussion, activity, or personal reflection) that provided insight into one or more of the following: self-awareness, self-management, social awareness, relationship skills, responsible decision-making, positive emotions, engagement, sense of meaning, and achievements. All +MHM experiences were led by the teacher in a standardized way, using prescriptive teacher dialogue and premade resources from the curriculum. +MHM experiences were designed to be delivered in 10 minutes, as this was the time allotment created for them.

To illustrate an example of +MHM, an academic lesson that explored harsh feedback for a game design linked itself to a discussion regarding the “purpose of emotions” and offered scripted strategies for controlling emotions calmly and professionally. An expanded example of an MHM lesson can be found in Multimedia Appendix 2.

### Measures

At baseline and post intervention (14 weeks), each school’s scholastic gaming teacher was emailed a link to the electronic study surveys. Upon receipt of the link, teachers distributed the surveys to each student enrolled in the study to complete within a standardized window of 5 days. All survey responses were deidentified through a school code plus a number that was selected by each student. Supervising teachers were directed to ensure no duplicate student numbers.

#### Demographic Characteristics

Students were asked to report their age; grade in school; number of years participating in scholastic gaming; race; gender; and if they identified as lesbian, gay, bisexual, transgender, queer (LGBTQ+). Grade in school was categorized into middle (grades 6 through 8) or high school (grades 9 through 12). Years of participation in scholastic gaming was categorized as “first year” versus “two or more years.” Race was asked by “Which do you identify with?” allowing participants to select one or more options. Race was classified as American Indian or Alaska Native, Asian, Black or African American, Hispanic or Latino, Native Hawaiian/Pacific Islander, White, more than one race, or prefer not to say or unknown. Gender was asked similarly to race, allowing participants to select male, female, or nonbinary. Students could also identify as LGBTQ+ or not LGBTQ+.

#### Self-Esteem

The Rosenberg Self-Esteem Scale (RSES), a validated scale consisting of 10 questions on a four-point Likert scale measured participant self-esteem at baseline and week 14 [23]. The total RSES score was calculated by summing responses to each question and reverse coding five questions that use negative phrases. Scores range from 0 to 30, with lower scores indicating lower levels of self-esteem. A score of less than 15 is considered low self-esteem [23,24]. A binary variable indicating if a student had low self-esteem (defined as RSES <15) was created.

### Data Analysis

Basic descriptive statistics (frequencies, means) were used to describe the study sample. Baseline demographic differences between the +MHM and –MHM groups were assessed using *χ*^2^/Fisher exact and *t* tests for categorical and continuous data, respectively. Changes in the RSES score from baseline to postintervention as a continuous variable were assessed via multivariable repeated measures linear regression, controlling for baseline imbalances between the study arms. A multilevel model was implemented to account for the clustering of individuals within each school. Subgroup analyses were conducted using multivariable repeated measures linear regression to examine if there was a differential impact on the MHM curriculum by race, gender, and sexual orientation. Subgroup analyses collapsed the following racial categories into an “additional races” category due to small sample sizes: American Indian and Alaska Native, Asian, Native Hawaiian or Pacific Islander, more than one race, or prefer not to say or unknown. Students who chose not to self-report their gender were excluded from the subgroup analyses related to gender. The association between the MHM curriculum and improvement of low self-esteem (RSES score <15) was assessed using multivariable logistic regression controlling for baseline imbalances between the study groups. All analyses were completed using SAS Version 9.4 (SAS Institute, Inc).

## Results

**Figure 1.**
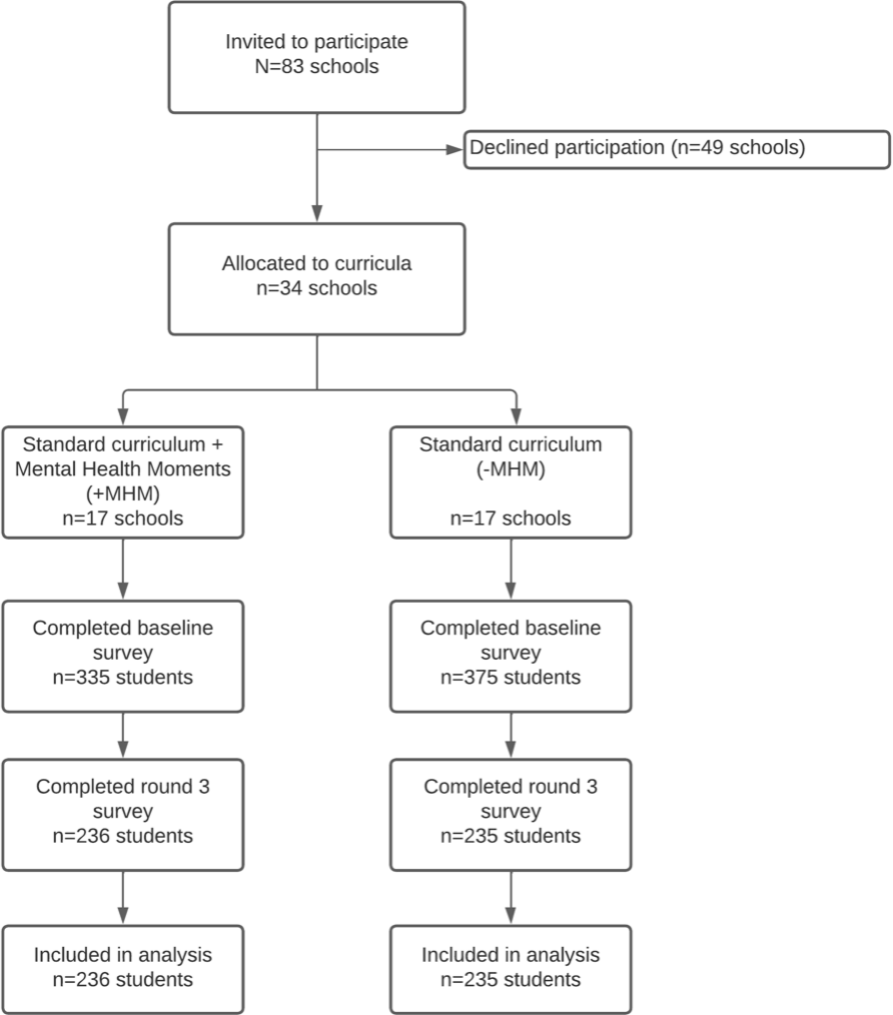
Study participation.

Among the 34 participating schools, 710 students enrolled in the study. Of those, 471 students completed all surveys (+MHM: n=236; –MHM: n=235) and were included in the analyses (Figure 1).

### Demographic Characteristics

The majority (n=353, 74.9%) of the 471 participants were enrolled in grades 9-12 at the time of the study, were male (n=387, 82.2%), and reported that this was their first year participating in scholastic gaming (n=429, 91.1%). About half (n=273, 58%) reported their race as White. The characteristics of students in each study group were balanced at baseline, except for age and race. The mean age of students in the +MHM arm was slightly less than that of the –MHM arm (14.9, SD 1.5 years vs 15.2, SD 1.8 years; *P*=.04). The distribution of self-reported race was even between the study groups, except for the +MHM group that included more students who reported their race as Asian than the –MHM group (19/236, 8.1% vs 9/235, 3.8%), and the +MHM group also had fewer students who reported their race as White than the –MHM group (125/236, 53% vs 148/235, 63%; Table 1).

**Table 1.** Baseline characteristics of study participants.

Characteristic		Total (n=471)	+MHM^a^ (n=236)	–MHM (n=235)	*P* value^b^
**Years of participation, n (%)**					.99
	First year	429 (91.1)	215 (91.1)	214 (91.1)	
	Two or more years	42 (8.9)	21 (8.9)	21 (8.9)	
**Grade level, n (%)**					.38
	Middle school	118 (25.1)	55 (23.3)	63 (26.8)	
	High school	353 (75.0)	181 (76.7)	172 (73.2)	
Age (years), mean (SD)		15.0 (1.6)	14.9 (1.5)	15.2 (1.8)	*.04* ^c^
**Race, n (%)**					*.047* ^d^
	American Indian or Alaska Native	16 (3.4)	5 (2.1)	11 (4.7)	
	Asian	28 (5.9)	19 (8.0)	9 (3.8)	
	Black or African American	49 (10.4)	25 (10.6)	24 (10.2)	
	Hispanic or Latino	76 (16.1)	43 (18.2)	33 (14.0)	
	Native Hawaiian/Pacific Islander	5 (1.1)	2 (0.9)	3 (1.3)	
	White	273 (58.0)	125 (53.0)	148 (63.0)	
	More than one race	19 (4.0)	14 (5.9)	5 (2.1)	
	Prefer not to say/unknown	5 (1.1)	3 (1.3)	2 (0.9)	
**Gender identification, n (%)**					.96^d^
	Female	59 (12.5)	31 (13.1)	28 (11.9)	
	Male	387 (82.2)	192 (81.4)	195 (83.0)	
	Nonbinary	21 (4.5)	11 (4.7)	10 (4.3)	
	Prefer not to say/unknown	4 (0.9)	2 (0.9)	2 (0.9)	
**LGBTQ+^e^, n (%)**					.53
	Yes	48 (10.1)	22 (9.3)	26 (11.1)	
	No	423 (89.8)	214 (90.7)	209 (88.9)	
Rosenberg Self-Esteem Scale score^f^, mean (SD)		17.9 (5.1)	18.0 (5.4)	17.9 (4.9)	.80
**Low Rosenberg Self-Esteem Scale score** ^g^ **, n (%)**					.98
	Yes	104 (22.1)	52 (22.1)	52 (22.0)	
	No	367 (77.9)	183 (77.9)	184 (78.0)	

a MHM: Mental Health Moments.

b *P* value from *χ*2 or *t* test.

c Italics indicate statistically significant differences between study groups at baseline.

d Fisher exact test.

e LGBTQ+: lesbian, gay, bisexual, transgender, queer.

f Rosenberg Self-Esteem Scale scores range from 0 to 30, with lower scores indicating lower levels of self-esteem.

g A Rosenberg Self-Esteem Scale score <15 indicates low self-esteem.

There were statistically significant differences in the study group, race, and gender identification between those who were included in the analyses versus those excluded due to not completing the follow-up assessment. Students enrolled in the +MHM group were more likely to be included in the analyses than those in the –MHM group, students who declined to report their race at baseline and those who reported their race as Black or African American were less likely to be included than other races, and students reporting their gender identity as male or nonbinary were more likely to be included than those who reported their gender as female or declined to report (Multimedia Appendix 3).

### Self-Esteem

#### Baseline

The mean RSES score among the entire study sample at baseline was 17.9 (SD 5.1), with 22.1% (104/471) falling into the low self-esteem category (RSES <15). +MHM and –MHM had similar mean self-esteem scores at baseline (18.0, SD 5.4 and 17.9, SD 4.9, respectively; *P*=.80), with 52 participants in each group having low self-esteem (Table 1).

#### Postintervention –MHM

Among –MHM participants who had low self-esteem at baseline (RSES <15), 54% (28/52) had self-esteem scores in the normal range (15 or greater) by the end of the study (Table 2). RSES as a continuous variable did not change over time in the –MHM group (baseline RSES 17.9, SD 4.9; postintervention RSES 17.9, SD 5.5; Table 3). Self-esteem scores increased slightly among –MHM students reporting their race as White, Hispanic/Latino, and the races collapsed into the additional races category. Among –MHM students reporting their race as Black or African American, self-esteem decreased from baseline to the end of the study. Self-esteem scores also decreased among –MHM students reporting their gender as male, female, and nonbinary (Table 4).

**Table 2.** Odds of low self-esteem improving to normal by the end of the study period. Results of logistic regression controlling for age and race. The analysis includes 104 students with Rosenberg Self-Esteem Scale score <15 at baseline.

	Low self-esteem^a^ improved^b^, n (%)		aOR^c^ (95% CI)
	Yes (n=60)	No (n=44)	
+MHM^d^ (n=52)	32 (61.5)	20 (38.5)	1.17 (0.68-2.01)
–MHM (n=52; reference)	28 (53.9)	24 (46.2)	1.0

a Abnormally low self-esteem defined as Rosenberg Self-Esteem Scale score <15.

b Improvement measured as a score ≥15 by the end of the study.

c aOR: adjusted odds ratio.

d MHM: Mental Health Moments.

**Table 3.** Changes in self-esteem scores over time. Results of repeated measures linear regression controlling for age and race.

		Rosenberg Self-Esteem Scale score^a^, mean (SD)^b^		*P* values^c^		
		Baseline	Week 14	Group^d^	Time^e^	Group × time^f^
**Study group**				.14	*<.001* ^*g*^	*.002*
	+MHM^h^	18.0 (5.4)	19.5 (5.6)			
	−MHM	17.9 (4.9)	17.9 (5.5)			

a Rosenberg Self-Esteem Scale scores range from 0 to 30, with lower scores indicating lower levels of self-esteem.

b Raw means for respondents at each time point.

c *P* values are based on model-based means for repeated measure analyses controlling for age.

d *P* value group: differences in self-esteem scores at baseline between study groups.

e *P* value time: differences in self-esteem scores between baseline and week 14.

f *P* value group × time: differences in change in self-esteem score over time by study group.

g Italics indicate statistically significant results.

h MHM: Mental Health Moments.

**Table 4.** Subgroup analyses of changes in Rosenberg Self-Esteem Scale scores over time by race, gender, and sexual orientation. Results of repeated measures linear regression controlling for age and race.

Subgroup and study group				Rosenberg Self-Esteem Scale scores^a^, mean (SD)^b^		*P* values^c^		
				Baseline	Week 14	Group^d^	Time^e^	Group × time^f^
**Race^g^**								
	**Black**					.51	.11	*.004^h^*
		+MHM^i^ (n=25)		17.7 (5.9)	20.3 (6.0)			
		–MHM (n=24)		20.6 (5.3)	17.6 (5.1)			
	**Hispanic/Latino**					.12	*.01*	.19
		+MHM (n=43)		16.6 (5.8)	18.1 (5.0)			
		–MHM (n=33)		18.4 (3.6)	18.7 (5.1)			
	**White**					*.04*	*.002*	*.046*
		+MHM (n=125)		18.1 (5.4)	20.0 (5.8)			
		–MHM (n=148)		17.5 (5.1)	17.9 (5.9)			
	**Additional races** ^j^					.08	.74	.50
		+MHM (n=43)		19.3 (4.6)	18.9 (5.2)			
		–MHM (n=30)		17.1 (3.3)	17.6 (3.6)			
**Gender^k^**								
	**Male**					*.046*	*.002*	*.03*
		+MHM (n=192)		18.5 (5.4)	18.0 (4.8)			
		–MHM (n=195)		19.8 (5.7)	18.2 (5.5)			
	**Female**					.79	.79	*.006*
		+MHM (n=31)		16.6 (5.1)	18.7 (5.0)			
		–MHM (n=28)		17.7 (5.4)	16.8 (5.4)			
	**Nonbinary**					.81	*.008*	.71
		+MHM (n=11)		13.9 (4.2)	14.5 (3.6)			
		–MHM (n=10)		16.9 (6.4)	16.5 (5.8)			
**Orientation**								
	**LGBTQ+** ^**l**^					.52	*.001*	.10
		+MHM (n=22)		13.5 (4.7)	18.8 (6.4)			
		–MHM (n=26)		14.3 (3.8)	15.7 (4.3)			
	**Not LGBTQ+**					.21	*.03*	*.006*
		+MHM (n=214)		18.5 (5.3)	19.6 (5.5)			
		–MHM (n=209)		18.3 (4.8)	18.2 (5.5)			

a Rosenberg Self-Esteem Scale scores range from 0 to 30, with lower scores indicating lower levels of self-esteem.

b Displays raw means for actual respondents at each time point.

c *P* values are based on model-based means for repeated measure analyses controlling for age.

d *P* value group: differences in self-esteem scores at baseline between study groups.

e *P* value time: differences in self-esteem scores between baseline and week 14.

f *P* value group × time: differences in change in self-esteem score over time by study group.

g Race subgroup analyses did not control for race.

h Italics indicate statistically significant results.

i MHM: Mental Health Moments.

j Additional races collapsed due to small sample sizes. Includes American Indian or Alaska Native, Asian, Native Hawaiian or Pacific Islander, more than one race, and prefer not to say/unknown.

k Four students who did not wish to disclose their gender were excluded from these analyses.

l LGBTQ+: lesbian, gay, bisexual, transgender, queer.

#### Postintervention +MHM

Among +MHM students who had low self-esteem at baseline (RSES <15), 62% (32/52) had self-esteem scores in the normal range (15 or greater) by the end of the study (Table 2). Self-esteem improved by about 8.3 percentage points from baseline to postintervention for the +MHM group (baseline RSES mean 18.0, SD 5.4; postintervention RSES mean 19.5, SD 5.6; Table 3). Among racial subgroups, self-esteem improved among +MHM students reporting their race as Black or African American, Hispanic/Latino, and White. Self-esteem decreased slightly among those falling into the additional races category. Results also displayed improvements in self-esteem among +MHM students reporting their gender as female and nonbinary. The largest improvement in self-esteem was among the LGBTQ+ subgroup, whose mean RSES score improved from 13.5 (SD 4.7) at baseline to 18.8 (SD 6.4) by the end of the study (Table 4).

#### Postintervention –MHM Versus +MHM

Results from the multilevel multivariable linear regression found that mean RSES score improvement over time was greater among the +MHM group (baseline: 18.0, SD 5.4; week 14: 19.5, SD 5.6) compared to the –MHM group (baseline: 17.9, SD 4.9; week 14: 17.9, SD 5.5; group × time; *P*=.002; Table 3). Additional subgroup analyses found that the +MHM intervention improved self-esteem more than the –MHM intervention among students identifying as African American or Black, White, male, female, and non-LGBTQ+ (see *P* values for group × time in Table 4).

Results of the multivariable logistic regression found that, among students with low self-esteem at baseline, the odds of reaching normal levels of self-esteem by week 14 were greater among students who were enrolled in +MHM compared to those in the –MHM group (adjusted odds ratio 1.17, 95% CI 0.68-2.01), although this finding was not statistically significant (Table 2).

## Discussion

### Principal Results

Our study found that the scholarly gaming curriculum produced a clinically relevant migration of participants from abnormally low self-esteem baselines to a normal range, and the +MHM group had greater self-esteem score improvement than the –MHM group. This suggests that the insertion of embedded preventive mental health (+MHM) into the scholarly gaming curriculum may amplify the well-being benefit of the scholarly gaming curriculum. Furthermore, the large sample size over one semester suggests delivering an embedded mental health curriculum within an academic course is feasible.

Independent of the macroscopic changes that embedded preventive mental health might offer, it’s equally important to discuss the impact this intervention made on higher-risk cohorts of adolescents, as mental health burdens are not carried equally.

### Implications for Adolescents at Higher Risk for Mental Health Threats

The Centers for Disease Control and Prevention reports that females; students of color; and individuals who identify as lesbian, gay, or bisexual are often at higher risk for mental health challenges throughout adolescence [25,26]. Subgroup analyses within our study revealed differences in the impact of the scholarly gaming curriculum (with or without MHM) between races, genders, and LGBTQ+ identification. These changes were magnified in the +MHM group, suggesting that embedded preventive mental health might offer some valuable improvements in self-esteem for several of the CDC-defined “higher risk” cohorts.

### Female Adolescents

Participants who identified as female and received +MHM experienced an increased trend in self-esteem from baseline to the end of the study when compared to those who identified as female and received –MHM. This positive impact linked to embedded preventive mental health (+MHM) is worth noting for adolescent advocates—something they might consider sharing with schools in their community, given that adolescent females displayed a 53% increase in emergency department visits for suicidal ideations in 2021 compared to 2019 [27].

### Students of Color

Racism can serve as a structural and social determinant of health that increases stress and negatively impacts mental health, making protective factors for mental health critically important to minoritized cohorts [28,29]. It is worth noting that Black and Hispanic/Latino participants who engaged with +MHM demonstrated increases in perceived self-esteem when compared to baseline values and the –MHM cohort for their race. It is possible that a curriculum that emphasizes student self-worth and provides an opportunity for peer connection as much as academic content may be beneficial for people of minoritized groups.

### LGBTQ+ Identification

Adolescents who identify as LGBTQ+ sometimes have trouble finding a supportive niche and friend groups in school [30]. This can be a detriment to mental health, as prior research demonstrates how inclusion in extracurricular activities boosts students’ perceptions of self-worth and confidence [31]. Enter scholarly gaming, an activity with no historical precedents and a nonthreatening environment for LGBTQ+ students potentially seeking a sense of belonging and friendship. The results demonstrated an increase in self-esteem scores for all LGBTQ+ participants post intervention. Furthermore, although not statistically significant due to sample size, students in the +MHM group who identified as LGBTQ+ had the lowest self-esteem scores at baseline and saw the largest improvement in self-esteem by the end of the study when compared to all other cohorts in the study. These findings may be related to benefits provided by the inclusive design of scholastic gaming and the impact of +MHM, suggesting that other school activities with a similar inclusive nature and embedded preventive mental health may benefit students who identify as LGBTQ+.

### Comparison With Prior Work

To the best of our knowledge, this study is one of the first to quantify the impact of embedded preventive mental health within standard curricula, as compared to traditional social-emotional learning, which is often taught independent of academic content. Equally important, this study made a deliberate choice to use scholarly gaming, a class arguably centered around technology and video gaming, as the conduit to +MHM. The intent was to demonstrate that if +MHM could offer benefits in a scholarly gaming curriculum, it might offer benefits in another academic curriculum. Future implementations of the +MHM curriculum will need to be scalable for non–scholarly gaming classes, perhaps through universal instructional strategies, to see if they offer benefits for participants.

### Limitations

The strengths of this study include its use of a longitudinal design, large sample size, and an intervention that was practical and affordable for schools to implement. Nevertheless, several limitations of this study should be considered. First, although the curriculum was prescriptive and standardized, we could not guarantee implementation validity, even with teacher training. Second, although the attrition rate was similar between both the +MHM and –MHM study groups, we could not definitively determine the causes of participant dropout. Third, the allocation of schools to the +MHM and –MHM groups used a matched pairs experimental design with the following key variables: student number, school number, Title 1 status, public versus private, and geographic distribution. While the nonrandom allocation pattern has the potential to introduce bias, we accounted for this by controlling for imbalances between the study groups at baseline and using multilevel modeling. Additionally, the LGBTQ+ categorization in our study was only able to classify a student as identifying as LGBTQ+ or not; we were unable to determine specific gender identities or sexual orientation. Further studies should consider more detailed identification. Finally, these findings may not be generalizable beyond students participating in a scholarly gaming curriculum. Future studies should evaluate the impact of the integration of +MHM into other curricula on student self-esteem.

### Conclusions

Scholarly gaming with +MHM, a tech-based course that seems to capture the interest of many students, might serve as a successful conduit for embedded preventive mental health curriculum, improving the self-esteem of participants. This is particularly relevant, as scholarly gaming curricula often reach some of the most vulnerable youth through school-based interventions. Furthermore, if an embedded preventive mental health curriculum offers benefits in a scholarly gaming course, it might offer benefits in other academic settings [32,33]. Adolescent advocates and researchers should be aware of this potential opportunity for rapidly expanding school-based interventions to support self-esteem.

## Supplementary material

10.2196/48401 Multimedia Appendix 1 Baseline school characteristics by inclusion in the study.

10.2196/48401 Multimedia Appendix 2 Example of a Mental Health Moments (+MHM) lesson.

10.2196/48401 Multimedia Appendix 3 Baseline student characteristics by inclusion in analyses.

## Abbreviations

CASEL: Collaborative for Academic, Social, and Emotional Learning
ISTE: International Society for Technology in Education
LGBTQ+: lesbian, gay, bisexual, transgender, queer
MHM: Mental Health Moments
RSES: Rosenberg Self-Esteem Scale
