# Supplementary material for: Effects of a Preventive Mental Health Curriculum Embedded Into a Scholarly Gaming Course on Adolescent Self-Esteem: Prospective Matched Pairs Experiment
Source: JMIR Serious Games. 2023 Dec 6;11:e48401. doi: 10.2196/48401 (PMC10721133; doi:10.2196/48401)
Supplement: Multimedia Appendix 1 [file games-v11-e48401-s001.doc]

**Appendix 1**. Baseline school characteristics by inclusion in study.

| Schools | All (n=83) | Agreed (n=34) | Declined (n=49) | *P-value* |
| --- | --- | --- | --- | --- |
| Students enrolled in district, median (IQR) | 8,781  (1,794 - 46,908) | 17,659  (2,964 –  46, 908) | 7,330  (1,661 - 42,618) | .36 |
| Title 1 School, n (%)     Yes     No | 39 (47.0)  44 (53.0) | 17 (38.6)  17 (43.6) | 27 (61.4)  22 (56.4) | .65 |
| Geography, n (%)     United States        Northeast        Southwest        West        Southeast        Midwest     Canada | 10 (12.1)  6 (7.2)  11 (13.3)  15 (18.1)  40 (48.2)  1 (1.2) | 2 (20.0)  2 (33.3)  6 (54.6)  1 (6.7)  22 (55.0)  1 (100.0) | 8 (80.0)  4 (66.7)  5 (45.5)  14 (93.3)  18 (45.0)  0 (0.0) | **.004*** |
| Public vs Private, n (%)     Public     Private | 69 (83.1)  14 (16.9) | 27 (39.1)  7 (50.0) | 42 (60.9)  7 (50.0) | .45 |
| Median household income (USD), median (IQR) | 58.825  (47,106 - 65,278) | 48,702  (47,016 - 59,948) | 53,261  (47,330 - 68,982) | .26 |
| Percent of children identifying as: (median, IQR)     White     Black     Hispanic/Latino | 70 (57 - 86)  6 (1 - 12)  9 (4 - 19) | 59 (59 - 85)  10 (1 - 12)  14 (7 - 19) | 71 (50 - 89.5)  5.5 (1 - 16.5)  8 (3 - 17) | .98  .62  .20 |
| Percent of children with health insurance, median (IQR) | 94.6  (92.2 - 97.6) | 95.7  (92.2 - 97.3) | 94.4  (92.2 - 97.6) | .52 |
| Percent of families below poverty level, median (IQR) | 19  (7.9 - 23.3) | 22.4  (8.4 - 23.3) | 17.55  (7.85 - 21.95) | .32 |
| Percent of families with Food Stamps/SNAP benefits, median (IQR) | 24.4  (10 - 25.4) | 25.1  (12.1 - 26.2) | 22.35  (9.9 - 26.2) | .57 |
| Percent of children with a disability, median (IQR) | 6.3  (4.2 - 7.2) | 7.2  (4.9 - 7.2) | 5.4  (4.15 - 7.2) | .20 |
| n=2 missing number of students enrolled in school district, Median household income, Race/ethnicity, Title 1 Status; n=3 missing Percent of children with health insurance; n=4 missing Percent of children with disability, Percent of families below poverty level, Percent of families with food stamps/SNAP benefits  *Fisher’s exact; **Bold** indicates statistically significant difference between schools who enrolled and did not enroll | | | | |
