# Supplementary figures and images for: Effects of a Preventive Mental Health Curriculum Embedded Into a Scholarly Gaming Course on Adolescent Self-Esteem: Prospective Matched Pairs Experiment
Source: JMIR Serious Games. 2023 Dec 6;11:e48401. doi: 10.2196/48401 (PMC10721133; doi:10.2196/48401)

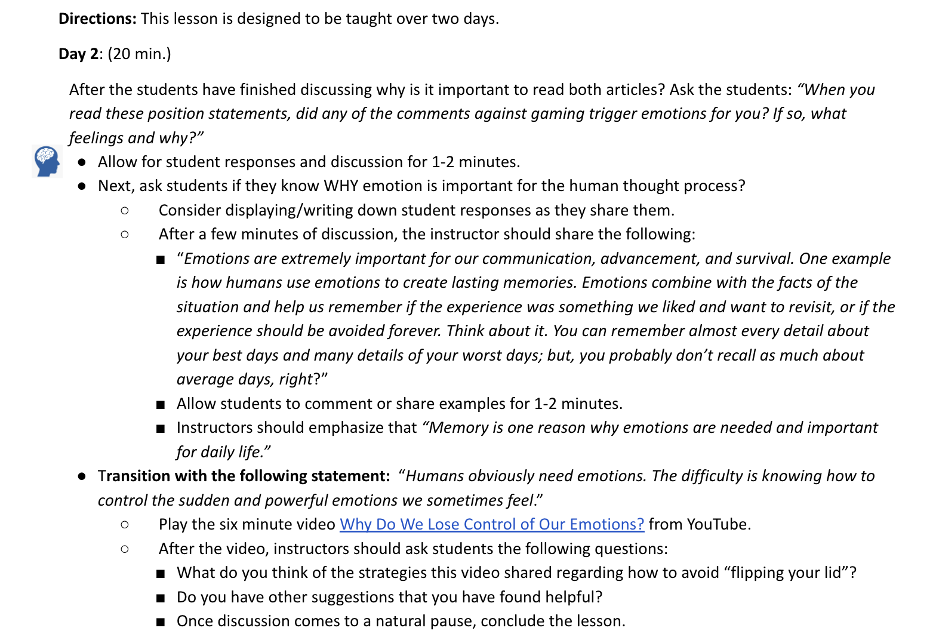

Supplement: Multimedia Appendix 2 [file games-v11-e48401-s002.png]
