# Supplementary material for: Effects of a Preventive Mental Health Curriculum Embedded Into a Scholarly Gaming Course on Adolescent Self-Esteem: Prospective Matched Pairs Experiment
Source: JMIR Serious Games. 2023 Dec 6;11:e48401. doi: 10.2196/48401 (PMC10721133; doi:10.2196/48401)
Supplement: Multimedia Appendix 3 [file games-v11-e48401-s003.doc]

**Appendix 3**. Baseline student characteristics by inclusion in analyses.

|  | All  (n=710) | Included (n=471) | Excluded (n=239) | *P*-value |
| --- | --- | --- | --- | --- |
| Study Group, n (%)     MHM     Control | 335 (47.2)  375 (52.8) | 236 (70.4)  235 (62.7) | 99 (29.6)  140 (37.3) | **.03** |
| Years of Participation, n (%)     First year     Two or more years | 648 (91.3)  62 (8.7) | 429 (66.2)  42 (67.7) | 219 (33.8)  20 (32.3) | .81 |
| Grade level, n (%)     Middle school     High school | 191 (26.9)  519 (73.1) | 118 (61.8)  353 (68.0) | 73 (38.2)  166 (32.0) | .12 |
| Age, mean (SD)* | 15.0 (1.8) | 15.0 (1.6) | 15.0 (1.8) | .99 |
| Race, n (%)*     American Indian/Alaska Native     Asian     Black or African American     Hispanic or Latino     More than one race     Native Hawaiian/Pacific Islander     Prefer not to say/unknown     White | 18 (2.5)  55 (7.8)  101 (14.2)  117 (16.5)  28 (3.9)  5 (0.7)  16 (2.3)  370 (52.1) | 16 (88.9)  28 (50.9)  49 (48.5)  76 (65.0)  19 (67.9)  5 (100)  5 (31.3)  273 (73.8) | 2 (11.1)  27 (49.1)  52 (51.5)  41 (35.0)  9 (32.1)  0 (0)  11 (68.8)  97 (26.2) | **<.001** |
| Gender identification, n (%)*     Female     Male     Non-binary     Prefer not to say/unknown | 107 (15.1)  567 (79.9)  27 (3.8)  9 (1.3) | 59 (55.1)  387 (68.3)  21 (77.7)  4 (44.4) | 48 (44.9)  180 (31.7)  6 (22.2)  5 (55.6) | **.02** |
| LGBTQ+*     Yes     No | 71 (10.0)  639 (90.0) | 48 (67.6)  423 (66.2) | 23 (32.4)  216 (33.8) | .81 |
| Rosenberg Self-Esteem Low, n (%)     Yes     No | 162 (22.8)  548 (77.2) | 104 (64.2)  367 (67.0) | 58 (35.8)  181 (33.0) | .51 |
| Rosenberg Self-Esteem Scale, mean (SD) | 18.0 (5.2) | 17.9 (5.1) | 18.2 (5.5) | .47 |

*p<0.05

^✢^Fisher’s exact
